# Endotoxemia related to cardiopulmonary bypass is associated with increased risk of infection after cardiac surgery: a prospective observational study

**DOI:** 10.1186/cc10051

**Published:** 2011-02-23

**Authors:** David J Klein, Francoise Briet, Rosane Nisenbaum, Alexander D Romaschin, C David Mazer

**Affiliations:** 1 Department of Critical Care and the Li Ka Shing Knowledge Institute, St. Michael's Hospital, University of Toronto, 4-054C Queen Wing, 30 Bond Street, Toronto, ON M5B 1W8, Canada; 2 Department of Anesthesia, St. Michael's Hospital, University of Toronto, 1-028e Shuter Wing, 30 Bond Street, Toronto, ON M5B 1W8, Canada; 3 Centre for Research in Inner City Health in The Keenan Research Centre, Li Ka Shing Knowledge Institute, St. Michael's Hospital, Dalla Lana School of Public Health, University of Toronto, 209 Victoria Street, Room 3-25B, Toronto, ON M5B 1T8, Canada; 4 Li Ka Shing Knowledge Institute, St. Michael's Hospital, Department of Laboratory Medicine and Pathobiology, University of Toronto, Room 2-006 Cardinal Carter Wing, 30 Bond Street, Toronto, ON M5B 1W8, Canada; 5 Department of Anesthesia and the Li Ka Shing Knowledge Institute, St. Michael's Hospital, Room 1-028e, Shuter Wing, 30 Bond Street, Toronto, ON M5B 1W8, Canada

## Abstract

**Introduction:**

Previous studies have documented a high frequency of endotoxemia associated with cardiopulmonary bypass (CPB). Endotoxemia may be responsible for some of the complications associated with cardiac surgery. The purpose of the study was to examine the prevalence of endotoxemia during cardiopulmonary bypass supported aortocoronary bypass grafting surgery (ACB) using a new assay, the Endotoxin Activity Assay (EAA), and explore the association between endotoxemia and post-operative infection.

**Methods:**

The study was a single center prospective observational study measuring EAA during the perioperative period for elective ACB. Blood samples were drawn at induction of anesthesia (T1), immediately prior to release of the aortic cross-clamp (T2), and on the first post-operative morning (T3). The primary outcome was the prevalence of endotoxemia. Secondary outcomes assessed included infection rates, intensive care unit (ICU) and hospital length of stay. An EAA of < 0.40 units was interpreted as "low", 0.41 to 0.59 units as "intermediate", and ≥0.60 units as "high".

**Results:**

A total of 57 patients were enrolled and 54 patients were analyzable. The mean EAA at T1 was 0.38 +/- 0.14, at T2 0.39 +/- 0.18, and at T3 0.33 +/- 0.18. At T2 only 13.5% (7/52) of patients had an EAA in the high range. There was a positive correlation between EAA and duration of surgery (*P *= 0.02). In patients with EAA ≥0.40 at T2, 26.1% (6/23) of patients developed post-operative infections compared to 3.5% (1/29) of those that had a normal EAA (*P *= 0.0354). Maximum EAA over the first 24 hours was also strongly correlated with risk of post-operative infection (*P *= 0.0276).

**Conclusions:**

High levels of endotoxin occur less frequently during ACB than previously documented. However, endotoxemia is associated with a significantly increased risk of the development of post-operative infection. Measuring endotoxin levels during ACB may provide a mechanism to identify and target a high risk patient population.

## Introduction

Since the beginnings of cardiopulmonary bypass (CPB) supported cardiac surgery in the 1950's, clinicians and surgeons have faced the challenge of balancing the desire to achieve optimal surgical results, while minimizing the consequences of exposure to cardiac bypass [1,2]. The inflammatory response to CPB has been implicated in many of the post-operative clinical problems that often occur in these patients including coagulopathy, respiratory failure, post-operative shock states, and multiple organ failure [3]. The pathophysiology of this inflammatory response is thought to involve a cascade of complement activation, activation of intrinsic and extrinsic coagulation systems, as well as activation of cellular components of inflammation and alterations in immune function [3]. Numerous cytokines and inflammatory mediators have been found to rise in patients exposed to CPB including IL-1β, IL-6, IL-8, TNF-α [4-6].

Endotoxin, or lipopolysaccharide (LPS), is a key component of the cell membrane of gram negative bacteria. Endotoxin is one of the most potent known activators of innate immunity and the inflammatory response in humans [7]. It was first identified in the serum of patients undergoing CPB over 20 years ago and proposed as a potential mediator of multiple organ failure and prolonged recovery after cardiac surgery [8]. Endotoxin is hypothesized to enter the systemic circulation during CPB by translocation of gut commensal microbes or LPS fragments across the intestinal mucosal barrier during the period of relative hypotension and hypoperfusion associated with extracorporeal support [9]. The prevalence of endotoxemia in patients on cardiopulmonary bypass has been estimated at up to 100% of ACB patients, although estimates are highly variable [8,9]. Endotoxin's true pathologic role during and after CPB, however, has been called into question as it has been difficult to correlate the degree of endotoxemia with adverse clinical outcomes. Several therapeutic strategies directed at minimizing or treating endotoxemia as a consequence of CPB including selective gut decontamination, pulsatile flow extracorporeal pumps, and LPS receptor inhibitors have been tried in patients without success [10-12]. In addition, the estimated prevalence of endotoxemia during cardiopulmonary bypass may be unreliable due to the challenges of assaying endotoxin *in vivo *using the traditional Limulus Amoebocyte Lysate (LAL) assay [13].

To clarify the role of endotoxemia, we investigated the prevalence of endotoxemia related to CPB in a cohort of patients undergoing elective cardiac surgery using the EAA for the measurement of endotoxin in blood. We further investigated the association between endotoxemia and the development of adverse clinical events including length of stay and development of post-operative infections.

## Materials and methods

### Study design

The study protocol was approved by the Research Ethic Board of St. Michael's Hospital. All subjects provided written informed consent. All patients were scheduled to undergo elective on-pump cardiac bypass surgery at St. Michael's Hospital in Toronto, Ontario, Canada. Patients were excluded if they had a history of recent myocardial infarction (less than one week), required redo surgery, emergent surgery or a surgical procedure in addition to ACB (for example, valve replacement). The study also excluded patients with other co-morbidities that involve significant active inflammation such as Crohn's disease, ulcerative colitis, HIV, a bone marrow disorder, active cancer, or significant renal insufficiency (creatinine >133 umol/L). Patients were enrolled between August 2005 and December 2007.

### Intra-operative management

All patients remained on their pre-operative medications as directed until the surgical date. Patients were anesthetized using a narcotic (sufentanil 1 to 2 μg/kg or fentanyl 10 to 20 μg/kg), a benzodiazepine (midazolam 0.1 to 0.15 mg/kg), isoflurane 0.2 to 1.5% and/or propofol 50 to 100 μg/kg/min, with muscle relaxation provided from rocuronium 0.6 to 1.0 mg/kg or pancuronium 0.1 mg/kg. Heparin was given to maintain an activated clotting time (ACT) >420 seconds during CPB. Bypass management included non-pulsatile pump flow of 2.4 L/minute/m^2 ^of BSA, mean arterial pressure 55 to 85 mmHg, temperature 33 to 35°C, and blood sugar 4 to 10 mmol/L. Myocardial protection was achieved with cold blood crystalloid cardioplegia, and a "hot-shot" (250 to 500 mL) was delivered just prior to the removal of the aortic cross-clamp. After separation from CPB, heparin was reversed with protamine (approximately 10 mg/1,000 units of heparin). Postoperatively, patients were managed in a specialized cardiovascular intensive care unit with standardized protocols for early extubation (two to four hours) and blood glucose control (target 5.1 to 8.0 mmol/L).

### Data collection

Data were collected by a dedicated clinical research nurse and included patient demographics, laboratory values including hematology, coagulation parameters, biochemistry, and liver and renal functions. Intra-operative data collected included duration of surgery and duration of bypass time up until the removal of the aortic cross clamp. We defined three time points for EAA collection: at the induction of general anesthesia (T1), at the time of removal of the aortic cross clamp after CPB (T2), and on the first post operative morning (T3). In addition, culture results were tracked and infection was established based on a clinical diagnosis. Length of intensive care unit stay and hospital stay were also tracked.

### Endotoxin activity assay

Endotoxin in whole blood was measured using the chemiluminescent endotoxin activity assay (EAA), as recommended by the manufacturer (Spectral Diagnostics, Toronto, ON, Canada). The methodology is described in detail elsewhere [14]. Briefly, samples of 50 μl of whole blood and appropriate controls were incubated in duplicate with saturating concentrations of an anti-lipid A IgM antibody, and then stimulated with opsonized zymosan. The resulting respiratory burst activity was detected as light release from the lumiphor, luminol, using a chemiluminometer (E.G. & G. Berthold Autolumat LB953, Wildbad, Germany). The LPS/anti-LPS complex primes the patient's neutrophils for an augmented response to stimulation with zymosan; by measuring basal (no antibody) and maximally stimulated (2,000 pg/ml LPS) responses in the same blood sample, the endotoxin activity of the test specimen is calculated by integrating chemiluminescence over time. Thus, the result is independent of white cell count or white cell responsiveness. Levels are expressed as endotoxin activity units, and represent the mean of duplicate determinations from the same sample. A level of less than 0.40 is defined as low, a level of 0.41 to 0.59 is defined as intermediately elevated, and a level of >0.60 is defined as highly elevated as per the recommendations of the manufacturer.

### Statistical analysis

Means, standard deviations and proportions were used to describe patients' characteristics. Group differences were examined using the chi-square or Fisher's exact test in binary variables, and the t-test or Wilcoxon rank sum test in continuous variables. We defined elevated EAA levels using three cut-off values: ≥0.40, ≥0.50, and ≥0.60. To account for correlations among repeated measures for each patient and for a few missing EAA values, change in EAA levels over time was evaluated using mixed models. Factors associated with the prevalence of elevated EAA pre-operatively (T1), at the time of the removal of the aortic cross-clamp (T2), and at 24 hours post-operatively (T3), were determined using generalized estimating equations. An unstructured covariance matrix was assumed in both models. All tests were two-sided and statistical significance was assumed for a *P*-value of ≤ 0.05. Analyses were performed using SAS version 9.2 (SAS Institute Inc., Cary, NC, USA).

## Results

### Patient characteristics

Fifty-seven patients were enrolled. One patient was excluded from the analysis because of lack of EAA data and 2 patients were excluded because of withdrawal of consent resulting in a sample size of 54 patients. Of these 54 participants, the mean age was 57.5 +/- 8.1, most were males (85.2%), 35.2% were current smokers, 46.3% had confirmed diabetes and 44.4% were obese (BMI ≥30) (Table 1). There were no statistically significant differences in patient characteristics between diabetic and non-diabetic patients. There was one death in the cohort due to cardiac arrest after a massive aspiration event.

**Table 1:** Patient characteristics (*n *= 54)

Characteristics	
% Males	85.2
Mean age (SD)	57.5 (8.1)
% Current smoker	35.2
% Diabetic	46.3
Mean (SD) BMI	30.34 (5.41)
% 18.50 to 24.99	14.8
% 25.00 to 29.99	40.7
% ≥30.00	44.5
Mean creatinine ( umol/L) (SD)	91.7 (22.4)
% Hypertension	77.8
% Hyperlipidemia	74.1
Median (IQR) duration of surgery (minutes)	190 (45)
Median (IQR) duration of cross-clamping (minutes)	56 (25)

BMI, body mass index; IQR, inter-quartile range; SD, standard deviation

### Endotoxin levels

**Figure 1:**
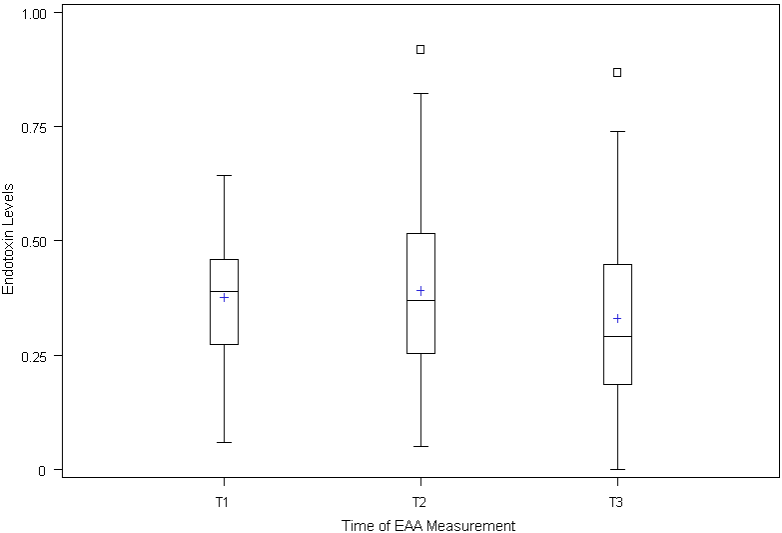
**Endotoxin Activity Assay measurement**. T1, induction of anesthesia; T2, at the time of removal of aortic cross clamp after CPB,; and T3, on the first postoperative morning. High EAA levels were rare at any time point.

The distribution of endotoxin levels at the three measured time points is represented in Figure 1. The mean EAA level at T1 was 0.38 +/- 0.14, at T2 was 0.39 +/- 0.19, and at T3 was 0.33 +/- 0.18. The prevalence of elevated EAA was at T1, T2, and T3 respectively: 48.1%, 44.2%, and 36.5% of patients had an EAA ≥0.40; 21.2%, 30.8%, and 15.4% had an EAA ≥0.50; and 5.8%, 13.5%, and 7.7% had an EAA ≥0.60. There were no significant changes in prevalence of elevated EAA over time. Prevalence of EAA ≥0.40 across all time points was similar for smokers and non-smokers (odds ratio 0.81 (CI: 0.35 to 1.88), and was not associated with age (odd ratio 1.01 (CI: 0.96 to 1.07)).

### Duration of surgery and cardiac bypass time

The median duration of surgery was 190 minutes (inter-quartile range (IQR) = 45 minutes). The median duration of cross clamp time was 56 minutes (IQR = 25 minutes). There was a significant correlation between EAA levels at T2 and the duration of surgery (Pearson correlation coefficient = 0.32, *P *= 0.02).

### Length of stay

The median length of hospital stay was seven days (IQR: six days), and 23 (43.4%) patients had a length of stay greater than seven days. One patient had a prolonged length of stay of 61 days associated with multiple complications. There were no statistically significant differences in length of stay for patients with EAA ≥0.40 versus patients with EAA < 0.40 at any point in time.

### Infections

**Figure 2:**
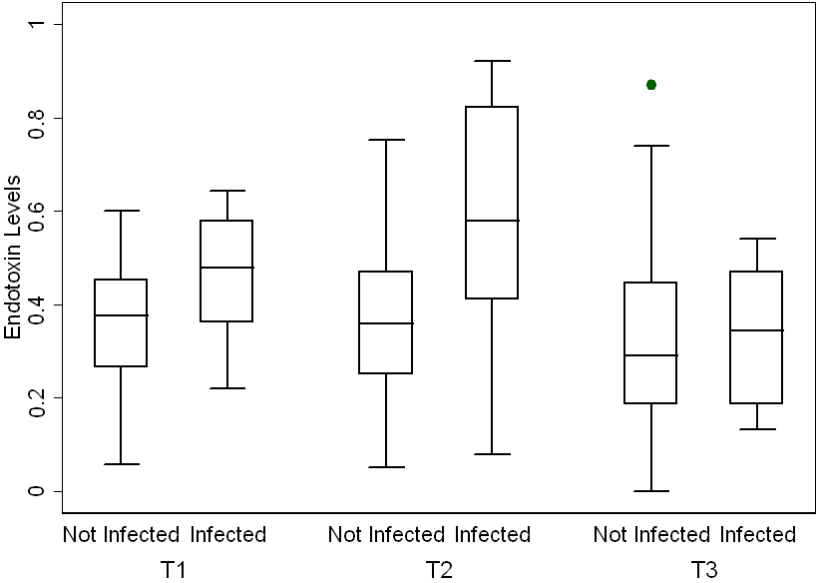
**Box plots for EAA levels in patients that developed post-operative infection versus those that did not**. Before (T1, *P *= 0.0796), during (T2, *P *= 0.0236) and after CPB (T3, *P *= 0.8203). Patients who went on to develop post-operative infection had significantly higher EAA levels at T2.

All patients underwent elective surgical screening procedures pre-operatively and none had clinical evidence of infection prior to surgery. A total of eight patients (14.8%) in the cohort developed postoperative infections. There were three cases of urosepsis, two cases of sternal wound infection or mediastinitis, three cases of cellulitis at the site of vein graft harvesting, and one case of pneumonia. One patient developed both urosepsis and wound cellulitis. EAA results for patients who developed infections versus those who did not are shown in Figure 2. In patients with EAA ≥0.40 at T2, 26.1% (6/23) of patients developed post-operative infections compared to 3.5% (1/29) of those that had a normal EAA (*P *= 0.0354). There was a non-significant trend for EAA levels at baseline to also be higher in patients that developed postoperative infections than in those that did not (mean (SD) = 0.46 (0.14) versus 0.36 (0.13), respectively). Differences were only statistically significant at T2 (median IQR) = 0.58 (0.41) and 0.36 (0.22), *P *= 0.0236. Similarly, the maximum EAA level across all the three time points was strongly associated with risk of subsequent infection (median IQR) = 0.62 (0.23) versus 0.45 (0.24) in the infection and no infection group, respectively (*P *= 0.0276).

## Discussion

In this study, we validate previous reports that endotoxemia occurs in patients exposed to CPB utilizing a novel independent method for measuring endotoxin *in vivo*. While our observed prevalence of endotoxemia at the end of CPB at 44.2% is similar to some reports, it is lower than many studies that have reported the frequency of endotoxemia related to CPB at as much as 100% [15,16]. Further, the incidence of patients having levels of endotoxin similar to those that might be observed in patients with severe sepsis (EAA >0.60), was quite low in our study, with only 7.7% of patients having this high level on this first post-operative morning [17]. There are several possible explanations for these observations. Prior studies have utilized the LAL assay. The LAL assay, however, has not proven to be dependable for quantitation of endotoxin in human blood or plasma due to interference from metals, amino acids, hormones, alkaloids, plasma proteins, electrolytes and antibiotics [18]. Dilution enhancement is a common problem with the LAL assay and this effect may be compounded in a cardiac bypass patient population due to changes in plasma composition during the course of, and following, the bypass procedure due to the use of cardioplegia solutions, crystalloids and hemodilution. In addition, we selected for study a relatively low risk cohort of patients going for cardiac surgery. All underwent elective procedures, those with advanced renal disease were excluded, as were those having complex valve operations or redo operations. Thus, exposure to prolonged periods of CPB was limited. Further, since the time of publication of previous reports, there have been substantial improvements in anesthetic techniques, perfusion practices, and in cardiopulmonary bypass circuits themselves [19]. These improvements likely have decreased the incidence of endotoxemia during CPB through a variety of mechanisms including decreased activation of coagulation factors and complement, improved tissue oxygen delivery and, therefore, decreased ischemia-reperfusion injury to the bowel, and shortened exposure time to the CPB circuit. Finally, the timing and frequency of sampling may influence our observations compared to previous reports such as the study by Boelke *et al.*, which observed that endotoxin levels peaked at reperfusion but remained quite elevated six hours post-operatively before decreasing to an only slightly elevated level on Day 1 [16].

Interestingly, we observed that a substantial number of patients presenting for elective cardiac surgery had small elevations in endotoxin levels before surgery. While active smoking has been associated with endotoxemia, we did not find a similar correlation in our cohort with subjective smoking status on history. However, we did not adjudicate the time of the patient's last cigarette [20]. Others have found elevations in endotoxin levels associated with chronic heart disease including severe heart failure [21,22]. It has been hypothesized that translocation of endotoxin from the gut in these cases contributes to edema and acute exacerbations via activation of the inflammatory cascade. We did not specifically measure left ventricular ejection fraction prior to surgery in our study. Further support to the validity of the observed levels of endotoxin preoperatively is the known presence of endogenous anti-endotoxin antibodies in patients going for cardiac surgery [23].

Infection is a common complication after cardiac surgery. The finding of a substantially increased risk of post-operative infections in patients who have endotoxemia after CPB is novel. Given the elective nature of the surgical patients and their extensive pre-operative screening, it is unlikely that they had occult infections prior to surgery or developed them intraoperatively. Rather, we suggest that perioperative endotoxemia results from translocation of endotoxin from gut commensal bacterial flora during CPB. Thus, this period of endotoxemia represents the first "hit" in a two "hit" model of risk. Faist *et al. *first used this "two hit" hypothesis to describe the increased risk of development of sepsis in patients after polytrauma [24]. Similar models have been described in other critical illnesses including burns [25]. Volk *et al. *have described this phenomenon as "immunoparalysis", whereby patients subjected to a first "hit" down-regulate HLA-DR4+ monocytes in response to an acute rise in inflammatory mediators including IL-8 and TNF-α [26]. These patients have been found to have an increased risk of postoperative infections. It has been hypothesized that this phenomenon may be linked to translocation of endotoxin [27]. It has been further suggested that immune monitoring in the postoperative period may be useful in identifying patients at risk [27]. Faist *et al. *have also described a pilot-clinical trial of GM-CSF to counter immunoparalysis [28]. Conversely, the finding of antibodies to endotoxin in patient's blood prior to gynecologic surgery has been found to reduce the risk of post operative infections [29].

Attempts to therapeutically target endotoxin in patients going for cardiac surgery have largely been disappointing. Strategies have included antagonists to the endotoxin Toll-like receptor 4 (TLR4), extracorporeal endotoxin removal systems, performance "off pump" cardiac surgery to eliminate CPB exposure, engineered anti-endotoxin monoclonal antibodies as well as other methods [10,30,31]. We hypothesize that these failures may in part be explained by our findings of a relatively lower prevalence of high amounts of endotoxin in CPB patients after surgery coupled with the failure of these studies to measure endotoxin during or after CPB and specifically target the sub-population of patients who develop endotoxemia.

Our study has a number of important limitations. First, we studied a relatively low risk patient population and thus had a small number of patients for the determination of "hard" clinical outcomes, such as infection or mortality. Validation of these finding in multiple centers in larger numbers of patients is also warranted. In addition, it has been suggested that hemodilution of endotoxin by the administration of endotoxin free crystalloid solutions during CPB may lead to an underestimation of "true" circulating endotoxin levels. Nevertheless, previous studies similarly did not correct for hemodilution and thus we did not to do so for comparative purposes. We are not aware of any validated correction factor for hemodilution for endotoxin levels with any assay as endotoxin exists in many forms and compartments *in vivo *and the impact of hemodilution on each of these is unknown. In addition, we did not measure other inflammatory markers and immune markers in our study.

## Conclusions

This study confirms, with us using an independent method, that endotoxemia occurs in some patients having cardiac surgery, although rarely at high levels. Importantly, endotoxemia at the conclusion of CPB is associated with a significant risk of the development of postoperative infections. Further research is necessary to assess whether a targeted strategy of rapid measurement of endotoxin levels coupled with a directed anti-endotoxin therapeutic strategy could improve patient outcomes.

## Key messages

• The prevalence of high levels of endotoxemia (as measured by the Endotoxin Activity Assay) in patients undergoing elective cardiopulmonary bypass supported aortocoronary bypass grafting surgery is uncommon compared to previous reports

• Endotoxemia correlates with the duration of surgery

• Patients who do have cardiopulmonary bypass associated endotoxemia are at a significantly increased eight-fold risk of developing post-operative infections

## Abbreviations

ACB: aortocoronary bypass grafting surgery; ACT: activated clotting time; CPB: cardiopulmonary bypass; EAA: Endotoxin Activity Assay; IL: interleukin; IQR: inter-quartile range; LAL: Limulus Amoebocyte Lysate Assay; LPS: lipopolysaccharide; T1: time of induction of anesthesia; T2: time immediately prior to release of the aortic cross-clamp; T3: time of blood draw on first post-operative morning; TLR4: toll-like receptor 4; TNF-α: tumour necrosis factor alpha

## Competing interests

ADR is a co-inventor of the Endotoxin Activity Assay. DJK and ADR have served as consultants to Spectral Diagnostics Inc. (Toronto, ON, Canada). All other authors declare that they have no competing interests.

## Authors' contributions

DJK designed the study, analyzed the data, and authored the manuscript. FB was involved in study design, data collection and analysis. RN was responsible for statistical analysis and contributed to the manuscript. ADR was involved in performing the assay and data analysis, and contributed to the manuscript. CDM was involved in study design and data analysis, and contributed to the manuscript. All authors reviewed and approved the final manuscript.
